# Psychometric evaluation of the Australian interprofessional socialisation and valuing scale: An invariant measure for health practitioners and students

**DOI:** 10.1371/journal.pone.0309697

**Published:** 2024-09-06

**Authors:** Bau Dilam Ardyansyah, Reinie Cordier, Margo Brewer, Dave Parsons

**Affiliations:** 1 Curtin School of Allied Health, Faculty of Health Sciences, Curtin University, Perth, Australia; 2 Department of Medical Education, Faculty of Medicine, Hasanuddin University, Makassar, South Sulawesi, Indonesia; 3 Department of Social Work, Education and Community Wellbeing, Faculty of Health and Life Sciences, Northumbria University, Newcastle upon the Tyne, United Kingdom; 4 Department of Health and Rehabilitation Sciences, Faculty of Health Sciences, University of Cape Town, Cape Town, South Africa; 5 St John of God Public and Private Hospitals Midland, Perth, WA, Australia; KSAU-HS: King Saud bin Abdulaziz University for Health Sciences, EGYPT

## Abstract

**Objectives:**

This study aimed to evaluate the psychometric properties of the Australian Interprofessional Socialisation and Valuing Scale (ISVS)-21 and provide an invariant measure for health practitioners and students to assess interprofessional socialisation.

**Methods:**

The COnsensus-based Standards for the selection of health Measurement INstruments (COSMIN) were used as guidelines. This research began with a key step: conducting a pilot study to assess content validity, a requirement of COSMIN for item development. The ISVS-21 has not yet been validated in Australia. Content validity checks ensure the developed items accurately represent the measured construct in the intended cultural context. In addition to conducting more comprehensive tests of psychometric properties compared to previous studies on ISVS-21, this paper introduces something new by evaluating the internal structure of the instrument involving measurement invariance and hypothesis testing for construct validity based on several assumptions related to interprofessional socialisation and values. An invariant measure validates the use of the Australian ISVS-21 on practitioner and student equivalently, allowing the comparison of outcomes at both levels.

**Results:**

The evaluation of content validity indicated that the items were relevant, comprehensible (practitioners and students had an agreement score of >70% for all 21 items), and comprehensive to the concepts intended to be measured. Structural validity confirms ISVS-21 Australia as unidimensional, with good internal consistency reliabilities, Cronbach’s *α* scores = 0.96 (practitioner) and 0.96 (student). Measurement invariance tests confirm ISVS-21 Australia is configural, metric and scalar invariance (ΔCFI ≤ 0.01) across the tested groups of practitioner and student, and therefore suitable for use by both cohorts in Australia. Age and length of work/study were discriminant factors for interprofessional socialisation in both cohorts; the professional background was a differentiating factor for practitioners but not for students. Hypotheses testing results support the COSMIN construct validity requirement for the measure, with 83.3% of assumptions tested accepted.

**Conclusion:**

The Australian ISVS has good psychometric properties based on evaluating the content validity, internal structure, and hypotheses testing for construct validity. In addition, Australian ISVS is an invariant measure for use by health practitioners and students and, therefore, confirmed as a quality measure to assess interprofessional socialisation for both cohorts in Australia.

## Introduction

The World Health Organization (WHO) calls for education and health systems to embed interprofessional education (IPE) in tertiary curricula for the future workforce and in continuing professional development for the current workforce to ensure health professionals have the competencies to be effective interprofessional collaborative practitioners [1]. In response to this call to action, Australia made some early progress on embedding IPE in health professional education at the tertiary level [2], progress that continues today [3].

Most IPE to date results from the leadership of local champions within universities [2, 4] and health service organisations [2, 5, 6] rather than national leadership. Australia is moving towards national leadership with the Australian Health Practitioner Regulation Agency (AHPRA), the national regulation agency covering 28 professions, having recently released a statement of intent on interprofessional collaborative practice (IPCP) [7]. AHPRA is committed to advancing interprofessional collaborative practice in education, training, clinical governance, and healthcare delivery. Through this initiative, the vision is to provide more effective, efficient, culturally sensitive, and patient-centered care while actively working to eliminate racism in healthcare. The ultimate goal is to enhance collaboration within the healthcare workforce, which leads to improved experiences and better health outcomes for individuals and other healthcare consumers. To achieve AHPRA’s vision for IPCP, they need high-quality IPE in health professional education that results in the development of core IPCP competencies. To measure the achievement of these competencies, valid and reliable instruments that can be used across the professional lifespan are needed (from pre-qualifying students to experienced health practitioners).

Several instruments have been used in Australian universities to measure the outcomes of interprofessional education, including the Interprofessional Socialization and Valuing Scale (ISVS), the Readiness for Interprofessional Learning Scale (RIPLS), the University of West England (UWE) instruments, and Curtin University’s Interprofessional Capability Assessment Tool (ICAT) [4]. Australia needs more instruments for measuring interprofessional-related outcomes that have been validated with an Australian population. Until recently, the only instrument validated in the country was the Collaborative Practice Assessment Tool (CPAT) [8]. Having Australian-validated measures will significantly benefit the advancement of interprofessional education and collaborative practice in the country.

### The Interprofessional Socialization and Valuing Scale (ISVS)-21

Interprofessional socialisation is critical in shaping a profession’s values and beliefs [9]. Historically, healthcare professionals were educated and socialised separately, leading to professional isolation. Successful interprofessional collaboration hinges on critical values such as shared goals, responsibility, leadership, teamwork, and respect for other professions, underscoring the vital role of interprofessional socialisation [9–11]. A tool to measure interprofessional socialisation and its associated values is indispensable. In particular, the ISVS-24 [12] and its refined version, the ISVS-21 [9], are among the instruments widely used in Australia. The ISVS-24 consists of three domains: Self-Perceived Ability to Work with Others (*Self-Confidence*; 9 items), Values in Working with Others (*Attitude*; 9 items), and Comfort in Working with Others (*Behavior*; 6 items). The Cronbach *α* scores for these domains range from 0.79 and 0.89. The refinement of ISVS-24 into ISVS-21 involved more than just removing three items. To develop the ISVS and its instrument variations (including the ISVS-9A and ISVS-9B), the author created an item pool consisting of 34 items. Several items were added and removed from the item pool during the refinement process. The ISVS-21 contained 11 items that were different from the ISVS-24 (these items were added during the refinement process, and eight new items from the ISVS pool). The ISVS-24 is considered the only measure to comprehensively assess multiple levels of interprofessional outcomes based on the Kirkpatrick adapted model (covers outcomes evaluation for Level 2a, attitudes/perceptions; Level 2b, knowledge/skills; and Level 3, behaviours [13].

Different to ISVS-24, the ISVS-21 is unidimensional and was found to have better psychometric properties than the previous ISVS-24 version, with a Cronbach’s *α*=0.988 (95% confidence interval of 0.985–0.991) [9]. The ISVS-21 is considered a reliable measure of interprofessional socialisation in both practitioners and students (the agreement factor score between the practitioner and the student datasets, r = 0.9986, 95% CI 0.9983–0.9988) [9]. The original ISVS-21 consisted of 21 positively written items.

Both the ISVS-24 and ISVS-21, have been used and adapted cross-culturally in many countries with practitioners [14, 15] and student participants [11, 16–29]. These interprofessional scales have been used in several Australian studies [4]; however, they have been mainly used with student participants [11, 17, 21, 27, 30, 31], with limited practitioner involvement [32]. To date, these instruments have not been validated for either students or practitioners in Australia.

A valid and reliable measure of interprofessional socialisation is needed to measure outcomes in research, and a psychometric evaluation is required to ensure the adopted items adequately reflect the measure intended in the original version [33–35]. As students may dislike some items highly endorsed by practitioners, and vice versa [8, 14, 27, 36]. Following the COnsensus-based Standards for selecting health Measurement Instruments (COSMIN) requirements, validation was conducted simultaneously with practitioner and student cohorts as the intended users of the instrument [34, 35].

Initially established in 2015, COSMIN is a psychometric framework with a robust quality rating system for determining the risk of bias in testing psychometric properties [34, 35]. COSMIN consists of an international multidisciplinary team of researchers with backgrounds in epidemiology, psychometrics, medicine, qualitative research, and healthcare who are experts in developing and evaluating outcome measures. COSMIN introduced a psychometric framework and taxonomy designed to tackle the inconsistent and conflicting use of psychometric terms in research. COSMIN allows for the systematic assessment of measures in two critical areas: the methodological quality of research and the quality of psychometric properties of measurements. COSMIN provides each psychometric property with independent measurement standards and criteria for ranking its quality. This method allows for subscale and overall measurement quality assessment, making COSMIN unique and, arguably, more advanced than other psychometric frameworks. Having separate quality scores for each psychometric property in a measure provides a more comprehensive basis for assessment. COSMIN is recommended as a framework for instrument developers and people identifying the most appropriate measure for their purposes.

### Interprofessional socialisation and values for construct validity

#### Age and length of practice

The collaboration and practice of interprofessional skills are influenced by age and length of experience [37, 38]. Graduate students generally exhibit stronger interprofessional skills than undergraduates, possibly due to their age and maturity [39]. Furthermore, a study found that a one-year fellowship significantly improved interprofessional skills compared to a one-semester IPE course, highlighting the importance of course duration in shaping comfort, value, and ability to collaborate with other professions [40]. Practitioners’ willingness and ability to collaborate with other team members are affected by their understanding of age and experience in teamwork [14].

The findings indicate that experience accumulates over time, reinforcing practitioners’ ability to understand the scope of practice [41–43]. Forming a professional identity and establishing moderate collaborative relationships typically takes at least six months, with initial exposure being a significant point [43, 44]. However, it is essential to note that practitioners’ abilities to collaborate cannot be generalised based solely on the length of their work period. Even a senior medical practitioner with extensive practice experience may show reluctance to share information and collaborate interprofessionally [37, 45].

#### Professional backgrounds

Studies have highlighted a gap in how the healthcare profession perceives the concepts and values of interprofessional collaboration. The medical profession has historically dominated healthcare systems [46–48]. Physicians’ relationships with other professions are characterised by a lack of trust in different professions, reluctance to delegate work, and reluctance to collaborate [43, 46–48]. In a study by Taylor et al., 2016, physician residents, particularly in internal medicine, displayed the lowest index scores for interprofessional collaboration [49]. Conversely, social workers tend to have the most positive perception of interprofessional collaboration compared to almost all other health professions, positioning them at the opposite end of the spectrum. Nurses fall in the middle, scoring higher than physicians but lower than other non-physician professions. This difference is attributed to social work education, which has long emphasised interprofessional collaboration skills within its curriculum [50]. Differences in perceptions regarding interprofessional collaboration are also significant at the student level, for example, between medical and nursing students and medical and pharmacy students [17].

Taking an interprofessional approach to patient care is a significant advance in healthcare. In the past, medical and dental training focused on independent practice, causing older generations of practitioners from these professional backgrounds to overlook the importance of working with other healthcare professionals in patient care [51, 52]. However, a study has also found that a person’s professional background does not necessarily determine their ability to work collaboratively with other professionals different to their own [14]. In fact, there is growing enthusiasm for creating more equitable positions in health services. A study in Australia revealed disagreements between healthcare practitioners and students regarding the dominant role of medical practitioners in decision-making, and patient care [8]. Participants voiced a strong desire for change and rejected two items related to the perception of physicians having an overly dominant role in interprofessional teams.

### Objectives

Validation of the new version of the ISVS-21 using both practitioners and students is crucial to ensuring a quality measure of interprofessional competencies across the domains of attitudes, knowledge, skills, and behaviours for the Australian context [13]. This study aimed to: (1) validate the ISVS-21 in Australia and (2) evaluate the psychometric properties of the validated instrument in terms of content validity, internal structure (i.e., structural validity, internal consistency reliability, and measurement invariances), and hypotheses testing for construct validity. In particular, an evaluation of the internal structure of the instrument was carried out to determine the invariance of the instrument across the groups tested (practitioners and students) to ensure the instrument’s reliability for use in both cohorts.

## Materials and methods

### Procedures

**Fig 1:**
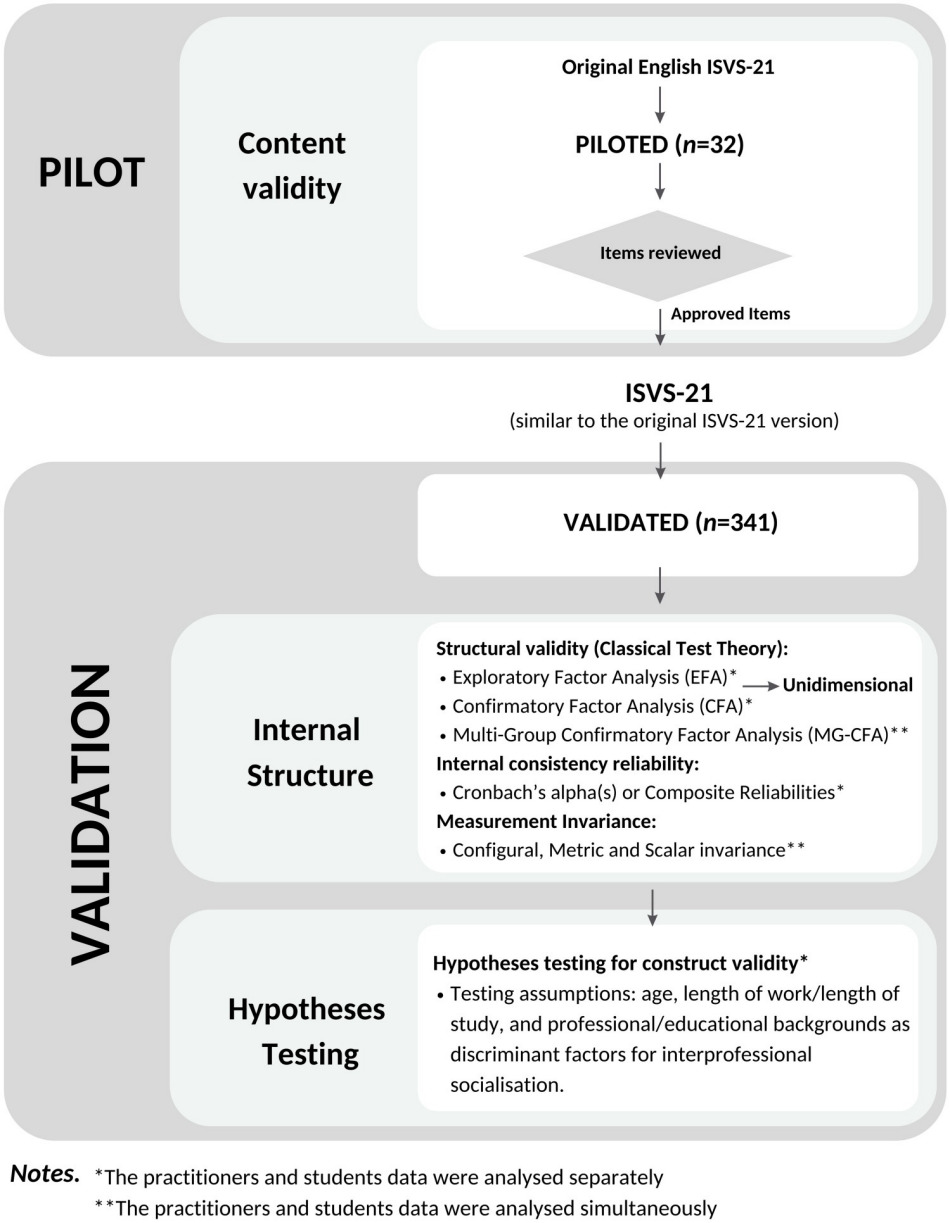
Study procedure.

We contacted the original authors of the instrument to advise them of our study validating the Interprofessional Socialisation and Valuing Scale (ISVS)-21 instrument for the Australian context. This study’s procedures, including instrument development requirements for data collection, analysis, and reporting, were based on the COnsensus-based Standards for the selection of health Measurement INstruments (COSMIN) taxonomy and standards of psychometric properties [33–35, 53]. The procedures for this study included pilot and validation studies. The phases are shown in more detail in Fig 1 below.

### Ethics statement

Ethics approval for this study was obtained from Curtin University Human Research Ethics Committee (HREC approval number: HRE2021–0274). The survey was distributed between 6 August 2021 and 15 May 2022. The invitation to participate included information about the survey and provided a Qualtrics [54] link that anyone interested could access. Participation in this research was voluntary, and all responses were anonymous.

### Sample population and size

The participants were purposively recruited to ensure optimal size and representation of the intended target population [34, 35]. The inclusion criteria were as follows: 1) Australian practitioners and students from any health professional or educational background, and 2) practitioners should have at least one year of experience collaborating within a healthcare team with other practitioners from different professional backgrounds, while students should have at least one year of experience working within a healthcare team with other students from different educational backgrounds. The minimum requirement for collaborative experience is one year, as the professional identity of health workers is estimated to be formed at least six months after the beginning of clinical exposure [44].

Potential participants for the pilot study were purposefully selected from the research team’s network, targeting individuals with expertise in interprofessional education and team collaboration. Participants’ agreement to participate was sought through invitations sent via email. The pilot study’s sample size was aimed at COSMIN’s ‘adequate’ requirements (i.e., 30–50 participants) for quantitative research and for a ‘very good’ sample size for the validation, with a 7 to 1 ratio of respondents to the number of items in each questionnaire and a minimum of 100 per sample (i.e., a minimum of 21*7 = 147 participants for each cohort of practitioner and student) [35].

An invitation for practitioners to participate in the validation study was sent to the relevant health professional peak bodies and associations in Australia. In addition, invitations were extended to health practitioners within the researchers’ network and snowballed to reach out to more participants. Invitations for student participation were sent through the university’s official communication platforms. Each participant was asked to provide their written consent before participating in the study. In addition, because providing consent was mandatory prior to initiating the survey, their consent was assumed based on the completion of the survey. The same participant inclusion criteria were used for the study’s instrument pilot and validation phase. Data collection for the pilot survey was completed four weeks prior to validation.

### Phase 1: Pilot study

The pilot study phases are described in alignment with COSMIN requirements to ensure that the instrument’s content validity adequately represents the underlying construct. Three aspects were evaluated in the pilot study [34, 35]; the items’ *relevance* and *comprehensibility*, and the *comprehensiveness* of the instrument.

#### Content validity

The ISVS-21 was piloted on Australian practitioners and students using a 5-point Likert scale (1=*strongly disagree* to 5=*strongly agree*). Participants were asked to rate whether each item was relevant to their experience (to assess item relevance) and whether each item was easy to understand (to assess item comprehensibility). Each participant who answered *disagree* or *strongly disagree* to the question regarding item comprehensibility was invited to explain their answer or suggest alternative phrasing to improve the clarity of the item. COSMIN recommendations were followed to assess the comprehensiveness of the instrument, an open-ended question was provided to identify whether participants felt any topics or items were missing from the instrument. Quantitative descriptive statistics were conducted using Statistical Package for the Social Sciences (SPSS) v26 [55]; while qualitative data were analysed using content analyses [56].

### Phase 2: Validation study

To analyse the instrument’s internal structure, the ISVS-21 was validated on the same target population as the pilot study [34, 35]. In the validation study, participants were asked to rate each item using the Likert scale used in the original instrument descriptors (0=*not at all*, 1=*strongly disagree*, 6=*strongly agree*) [9].

Quantitative descriptive statistics were applied to investigate the internal structure of psychometric properties of the Australian ISVS-21, including Exploratory Factor Analysis (EFA), using SPSS v26; Confirmatory Factor Analysis (CFA) and Multi-group Confirmatory Factor Analysis (MG-CFA), and measurement invariances were estimated using Analysis Movement of Structure (AMOS) v24 [57]. The analysis only includes data with a response rate of >75%, and missing data was replaced with the mean value. First, we tested to determine whether the data was suitable for factor analysis. Once we have qualified results, we can proceed with EFA and CFA. Kaiser-Meyer-Olkin (KMO) and Bartlett’s Test of Sphericity were used to determine the suitability of datasets for factor analysis [58]. To assess model fit, the following COSMIN criteria for good model fit indices were used to report on model fit throughout this study: a comparative fit index (CFI), Tucker-Lewis index (TLI) or equivalent measure >0.95, OR root mean square error of approximation (RMSEA) <0.06, OR standard root mean square of residual (SRMR) <0.08 [33]. The chi-square minimum difference function (CMIN/df) is expected to be between 1 and 3, with a score <5 regarded as acceptable.

#### Structural validity

The factor structure of the Australian ISVS was carried out in stages through Exploratory Factor Analysis (EFA), Confirmatory Factor Analysis (CFA) and Multi-group Confirmatory Factor Analysis (MG-CFA). Each stage is described in detail below.

Exploratory factor analysis was conducted to explore the constructs underlying the Australian ISVS before performing a confirmatory factor analysis [59]. The practitioner dataset was analysed separately from the student dataset. As stated earlier, the ISVS-21 was confirmed to be unidimensional from studies in several other countries [9, 14, 15]. To confirm unidimensionality, three decision rules were applied. Firstly, exploratory factor analysis dimension reduction with Kaiser’s criteria with eigenvalues greater than 1 [58] was used to determine the number of factors and the percentage of cumulative variance. If the first factor explains >40% of the variance of the population tested [60] or when the eigenvalue of the first factor is at least five times higher than that of the second factor [61], unidimensionality is confirmed. Secondly, a scree test [58] was conducted, which analyses the factorial structure following an eigenvalues plotline. The number of potential factors for a given data set can be determined by calculating the number of factors above the breakpoint in the plotline. Thirdly, based on correlation reliability checks with Cronbach’s *α* and average inter-item correlation scores [33, 62]. The higher the correlation between items, the higher the Cronbach’s *α* score; where a value of 0.70 is acceptable, a value >0.80 is considered high; a value >0.95 is undesirable as it may suggest item redundancy rather than homogeneity [33, 34, 63]. The average inter-item correlation was expected to be between 0.30 to 0.50.

Confirmatory factor analysis was performed to confirm the exploratory factor analysis results [58, 59]. CFA began by confirming the one-factor model for all 21 items. This confirmatory analysis was performed separately for each dataset, starting with the practitioner dataset, the results of which were used as a calibrator to inform the structure of the student model. The model’s goodness-of-fit indices (the CMIN/df, CFI, SRMR, RMSEA, *p*-value), composite reliability (CR), and average variance extracted (AVE) were analysed to assess the model fitness. These data profiles were used to determine whether the tested one-factor model was acceptable, needed improvement, or should be rejected. If required, problematic items were carefully removed one at a time during the CFA refinement process. Additionally, error terms covariance with a Modification Index (MI) >20 were identified [64]. Following the removal of each item and/or the application of one or more MI correlations, the fitness indices, CR, and AVE were recalculated each time. Confirmatory factor analysis was expected to confirm a good final model for both datasets to pass the multi-group factor analysis. This final factorial analysis was undertaken with both datasets simultaneously. The factor structures set as equal across tested datasets during MG-CFA to enable the rating of the quality of the summary score [33]. If MG-CFA shows a good model fit, measurement invariance can be conducted.

#### Internal consistency reliability

Internal consistency refers to the relationship between variables in measuring a similar construct that underlies the development of a domain, with distinctive domains indicated with good internal consistency and average variance extracted [33, 63]. At this stage, because the composite reliability value also considers the variable loading factor, the composite reliability score was used as a reference to measure the consistency of internal reliability. Similar cut-off values apply to composite reliability and Cronbach’s *α* scores [33, 63]. The average variance extracted was also calculated to estimate the discriminant validity of the domain; an average variance extracted >0.5 was expected [63].

#### Measurement invariances

Further analysis was conducted to ensure the equivalence of the two cohorts with invariant measurements [65, 66]. Both datasets were analysed simultaneously with configural, metric, and scalar invariant tests. Invariant measurement is a tiered test, where the next stage cannot be carried out if the proposed structural model does not provide a good model fit at the previous stage [65, 66]. As previously stated, the standard for good model fit follows the COSMIN guidelines [33]. During the invariant tests, the comparative fit index value was anticipated to decrease due to the imposition of some constraints on the model, but the decrease was expected to be ≤ 0.01 to confirm an invariance [65]. Invariance is confirmed when the difference in the comparative fit index (ΔCFI) ≤ 0.01 between the two tested measures [65].

### Hypotheses testing

Several elements are believed to influence the construct of interprofessional socialisation, including gender, age, length of service, and professional/educational backgrounds [37, 38, 41]. Therefore, COSMIN’s requirement for hypotheses testing was based on validating the assumptions associated with these elements. The hypotheses proposed were as follows:

**H1.A.** Age is a discriminant factor for interprofessional socialisation for health practitioners. Five age groups were created: 21–30 years, 31–40 years, 41–50 years, 51–60 years, and 61–70 years.
**H2.A.** Length of work is a discriminant factor for interprofessional socialisation for health practitioners. Seven groups of service length were created: 1–2 years, 3–5 years, 6–10 years, 11–15 years, 16–20 years, 21–30 years, and 31–40 years.
**H3.A.** Professional background is not a discriminant factor for interprofessional socialisation for health practitioners. There were 15 groups of professionals: dentist, nutritionist, medical practitioner, midwife, nurse, occupational therapist, optometrist, pharmacist, physiotherapist, podiatrist, psychologist, public health, radiographer, social worker, speech pathologist.
**H1.B.** Age is a discriminant factor for interprofessional socialisation for health students. Four age groups were created: 18–24 years, 25–29 years, 30–34 years, and 35–40 years.
**H2.B.** Length of study is a discriminant factor for interprofessional socialisation for health student length of study for health students. Four groups of length of study were created: 1–2 years, 3–4 years, 5–6 years, and 7–8 years.
**H3.B.** Discipline background is not a discriminant factor for interprofessional socialisation for health students. There were 13 student courses: dentistry, exercise and sports science, exercise physiology, health promotion and sexology, medicine, nursing, nutrition and dietetics, occupational therapy, pharmacy, physiotherapy, psychology, social work, and speech pathology.

## Results

### Phase 1: Pilot study

A total of 32 health practitioners (*n*=23) and students (*n*=9) completed the pilot study. Practitioners were aged between 21 and 60 years (*M*=39.3; *SD*=8.7); most were female (*n*=18, 78.3%) from eight different health professional backgrounds, with nurses being the largest group (*n*=11; 47.8%). Practitioners’ length of service in interprofessional collaborative practice settings varied from 1 to 30 years (*M*=9.2; *SD*=8.5). Students were aged 18 to 40 years (*M*=28.1; *SD*=7.3). All were female (*n*=9) and from five different educational backgrounds, with occupational therapy being the largest group (*n*=5; 55.6%). Students’ length of study varied from 1 to 4 years (*M*=3.0; *SD*=0.9). All students had experience working in teams in an actual healthcare industry, such as aged care, disability care, private clinics or hospitals. Detailed information regarding the pilot participants is provided as a supplementary (S1 Table).

#### Content validity

When asked about the *relevance* of the items, practitioners and students had an agreement score of >70% for all 21 items, indicating all items were considered relevant by health practitioners and students. Therefore, all 21 items were included in the validation (practitioners, *Md*=97.0, *n*=23; students, *Md*=93.0, *n*=9). Three items were rated *disagree* by practitioners (8.6%) and students (22.4%), which were ISVS6 (comfortable being the leader), ISVS7 (comfortable in speaking out), and ISVS1 (aware of preconceived ideas). The lowest minimum agreement score was for ISVS6 (comfortable being the leader)=78.3%.

When asked about the *comprehensibility* of the items, practitioners and students had an agreement score of >70% for all 21 items, indicating both cohorts understood the instructions, items, and response options as intended (practitioners, *Md*=97.0, *n*=23; students, *Md*=97.0, *n*=9). Nevertheless, the comprehensibility of four items, ISVS2 (better appreciation for using a common language), ISVS4 (able to share and exchange ideas), ISVS5 (enhanced perception of engaging in interprofessional practice), and ISVS15 (comfortable clarifying misconceptions) were each rated as *disagree* by practitioners (8.7%) and students (22.2%). The alternative wording suggested by the participants for the four items was brought to the research team’s panel meeting for further review. As the original instrument words and phrases were deemed more appropriate and better representing the items’ context than the suggested phrasing, no changes were made; the original version of all items was retained. No comments were provided when participants asked about missing concepts in the instrument, indicating practitioners and students considered the *comprehensiveness* of the instrument to be high (i.e., all key concepts were included). Due to the minimum concern raised by the participants during the pilot, one trial was deemed sufficient.

### Phase 2: Validation study

The practitioner cohort comprised 134 participants who were mainly females (*n*=107, 79.9%). Practitioners’ ages ranged from 21 to 70 years (*Md*=104.0; *n*=134), with the most common age group being 31–40 years (*n*=56, 41.8%). Practitioners’ length of service ranged between 1 and 40 years (*Md*=104.0; *n*=134), with 6–10 years of service being the most common range (*n*=31, 23.1%). The three most common professional groups were occupational therapy (*n*=30, 22.4%), speech pathology (*n*=23, 17.2%), and nursing (*n*=18, 13.4%). The student cohort comprised 207 participants who were mainly females (*n*=160, 77.3%). The students’ ages ranged between 18 and 44 years (*Md*=100.0; *n*=207), and the most common age range was 18–24 years (*n*=150, 72.5%). Students’ length of study within an interprofessional education and collaborative practice environment varied from 1 to 8 years (*Md*=100.0; *n*=207), with 1–2 years of studying within that context being the highest range (*n*=132, 63.8%). The three most common study courses were medicine (*n*=31, 15%), nursing (*n*=30, 14.5%), and speech pathology (*n*=23, 11.1%). All students involved had experience working in teams in actual healthcare industries, such as aged care, disability care, private clinics, or hospitals; length of working experience from 1 to 4 years (*Md*=100.0, *n*=207). More detailed information regarding participant characteristics is presented in Table 1.

**Table 1:** Participants characteristics.

Practitioners			Students		
Number of participants	n = 134		Number of participants	n = 207	
Demographics	Frequency (%)	Median	Demographics	Frequency (%)	Median
**Gender**					
Male	26 (19.4%)	96.5	Male	42 (20.3%)	101.0
Female	107 (79.9%)	104.0	Female	160 (77.3%)	100.0
Other	1 (0.7%)	106.0	Other	5 (2.4%)	104.0
**Age**					
21–30 years	31 (23.1%)	93.0	18–24 years	150 (72.5%)	100.5
31–40 years	56 (41.8%)	104.5	25–29 years	31 (15.0%)	103.0
41–50 years	26 (19.4%)	102.5	30–34 years	15 (7.2%)	107.0
51–60 years	15 (11.2%)	112.0	35–40 years	11 (5.3%)	83.0
61–70 years	6 (4.5%)	110.0			
**Length of work/Length of study**					
1–2 years	22 (16.4%)	93.0	1–2 years	132 (63.8%)	100.0
3–5 years	18 (13.4%)	94.0	3–4 years	63 (30.4%)	102.0
6–10 years	31 (23.1%)	102.0	5–6 years	10 (4.8%)	91.5
11–15 years	19 (14.2%)	105.0	7–8 years	2 (1.0%)	117.5
16–20 years	12 (9.0%)	113.5			
21–30 years	21 (15.7%)	105.0			
31–40 years	11 (8.2%)	109.0			
**Professional\Educational Backgrounds**					
Dentist	2 (1.5%)	72.5	Dentistry	11 (5.3%)	103.0
Nutritionist	3 (2.2%)	104.0	Exercise and sports science	3 (1.4%)	89.0
Medical practitioner	10 (7.5%)	110.5			
Midwife	3 (2.2%)	88.0	Exercise physiology	1 (0.5%)	63.0
Nurse	18 (13.4%)	106.0	Health promotion and sexology	11 (5.3%)	109.0
Occupational therapist	30 (22.4%)	102.5			
Optometrist	6 (4.5%)	90.0	Medicine	31 (15.0%)	100.0
Pharmacist	15 (11.2%)	98.0	Nursing	30 (14.5%)	103.0
Physiotherapist	7 (5.2%)	123.5	Nutrition and dietetics	10 (4.8%)	92.0
Podiatrist	2 (1.5%)	77.0	Occupational therapy	20 (9.7%)	104.0
Psychologist	4 (3.0%)	100.0	Pharmacy	22 (10.6%)	97.5
Public health	4 (3.0%)	105.0	Physiotherapy	12 (5.8%)	96.5
Radiographer	3 (2.2%)	113.0	Psychology	21 (10.1%)	104.0
Social worker	4 (3.0%)	117.5	Social work	12 (5.8%)	102.5
Speech pathologist	23 (17.2%)	86.0	Speech pathology	23 (11.1%)	102.0

With a total of 134 health practitioners and 207 students participating in the validation study, the study’s intended target population was fulfilled. COSMIN’s ‘adequate’ sample size (at least 5 times the number of items and a minimum of 100) was met for the practitioners, and ‘very good’ sample size (7 times the number of items and a minimum of 100) for students [34, 35]. The two datasets were confirmed suitable for factor analysis with Kaiser-Meyer-Olkin (KMO) 0.93 (practitioners) and 0.95 (students), respectively. Bartlett’s Test of Sphericity for both datasets had values of *p* <0.001, indicating suitability for factor reduction analysis. Results from the Kolmogorov-Smirnov normality test indicated evidence of non-normality (*p* <0.05 in both datasets). No significant outliers and no missing data were identified in both datasets.

#### Exploratory factor analysis

EFA was initiated with dimension reduction analysis set to maximum likelihood extraction based on eigenvalues greater than 1 and varimax rotation. Four potential factors were identified in the practitioner dataset and three in the student dataset, with a total variance explained in the first component being 53.8% and 59.4%, respectively. The assumption of factorial numbers based on Kaiser’s criteria with eigenvalues greater than one is presented in Table 2.

**Table 2:** Numbers of factorial structure based on Kaiser’s criteria.

Total Variance Explained							
Practitioner dataset				Student dataset			
Factor	Initial Eigenvalues			Factor	Initial Eigenvalues		
	Total	% of variance	Cumulative %		Total	% of variance	Cumulative %
1	11.30	53.8	53.8	1	12.48	59.4	59.4
2	1.48	7.1	60.8	2	1.34	6.4	65.8
3	1.28	6.1	67.0	3	1.07	5.1	70.9
4	1.00	4.8	71.7				

As presented in Table 2, the unidimensionality of the Australian ISVS is confirmed based on: (1) the first factor explaining >40% of the variance of the population tested in both datasets (practitioner = 53.8%; student = 59.4%) [60] and the total eigenvalue of the first factor being at least five times higher than that of the second factor in both datasets (practitioner 11.30/1.48 = 7.6 times higher; student 12.48/1.34 = 9.3 times higher) [61]; (2) the scree plots generated for both datasets indicated that the (imaginary) red line that separates the breakpoints in the two plots leaves only one dot above the line; and 3) all 21 items demonstrated strong internal consistency reliability with Cronbach’s *α* scores of 0.96 and 0.96 and average inter-item correlations between items of 0.51 and 0.57 for the practitioner and student datasets, respectively. These findings strongly supported the Australian ISVS factorial structure as unidimensional, resembling the original instrument factorial structure [9].

#### Confirmatory factor analysis

**Fig 2:**
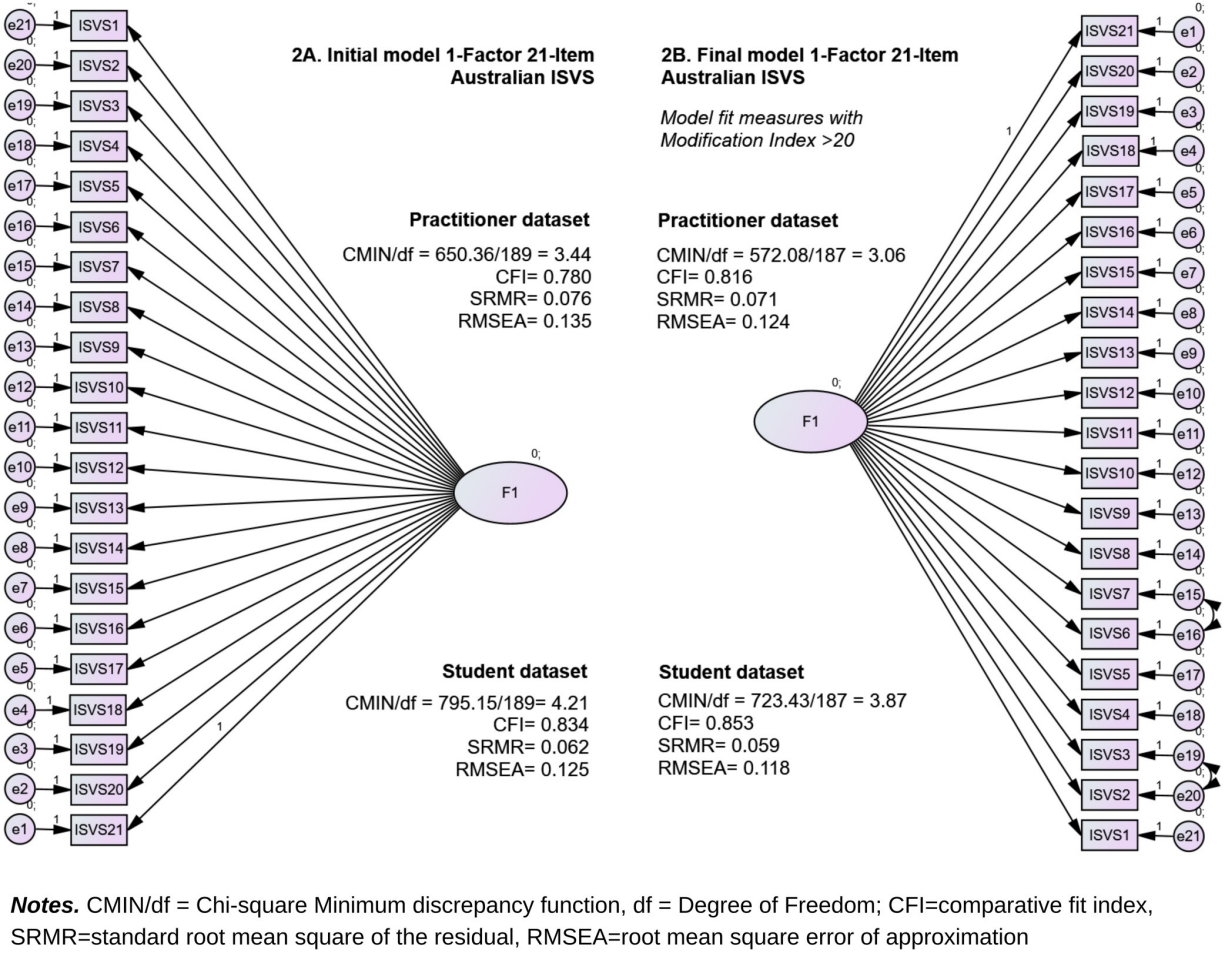
The initial (2A) and final (2B) structural models for the Australian ISVS.

CFA was conducted to further confirm a 1-factor 21-item solution for the Australian ISVS for both datasets. All 21 items for both cohorts showed good loading estimates >0.50 (ranging from 0.53—0.82 for practitioners and 0.64—0.85 for students), with a critical ratio (CR) >1.96 at *p* <0.001. These results indicated that each item met the validity requirements and reflected the unidimensional construct of the instrument. Sorted sequentially, ISVS1 (aware of preconceived ideas), ISVS2 (better appreciation for using a common language), and ISVS3 (enhanced awareness of own role) had the lowest estimated loading on practitioners. For students, ISVS2 (better appreciation for using a common language), ISVS6 (comfortable being the leader), and ISVS1 (aware of preconceived ideas) had the lowest estimated loading. The initial model tested with confirmatory factor analysis is shown in Fig 2A. Using this model, the two datasets were tested separately, using the practitioners as the calibrator. The results indicated a ‘good’ fit to the COSMIN model by the SRMR (practitioner SRMR = 0.076; student SRMR = 0.062) and acceptable CMIN/df with *χ*^2^(189)=650.36, CMIN/df = 3.44 for practitioners, and *χ*^2^(189)=795.15, CMIN/df = 4.21 for students. The CFI and RMSEA were poor in both datasets, with *p* <0.001 indicating a discrepancy between the data and the proposed models.

Several covariances were identified with the Modification Index (MI)>20, indicating the possibility of items overlapping. Seven significant cases were identified with MI>20 for practitioners. Notably, two of these cases related to error terms for ISVS1, ISVS2, ISVS3, and ISVS6, with the error terms covariance between ISVS2 and ISVS3 showing the highest MI (45.14 with a parameter change of 0.40). For students, ten cases were found with MI>20. Three cases involved error terms for ISVS3 and ISVS2, while two involved ISVS7. The highest MI (43.18 with a parameter change of 0.62) was observed in the covariance between the error terms for ISVS6 and ISVS7. Correlating the error terms of interest (ISVS2 and ISVS3, as well as ISVS6 and ISVS7) highlights areas for focused attention and potential enhancements of the model without the need to remove any item.

Alternative iterations were performed to improve the model fit by applying one or more covariances representing the MI findings [64]. As predicted, the most improved model fit was obtained by generating covariances with the most significant chi-square improvement and involved error terms of the same items in both cohorts, which were error terms between ISVS2 (better appreciation for using a common language) and ISVS3 (enhanced awareness of own role); and correlating between error terms for ISVS6 (comfortable being the leader) and ISVS7 (comfortable in speaking out). By correlating the error terms involving ISVS2 and ISVS3, as well as ISVS6 and ISVS7, the model fit indices for both data sets significantly improved. Fig 2 shows changes in fit indices of both datasets’ initial and final models.

As part of the iteration process, we conducted tests to analyse whether removing ISVS1 or ISVS6, or both ISVS1 and ISVS2, along with applying one or more correlations to error terms with MI>20 in both data sets, would significantly improve the model. Our goal throughout this iterative process is to retain as many items as possible. After excluding ISVS1 from the practitioner dataset, the CFI increased by 0.025 (from 0.780 to 0.805), and the SRMR decreased by 0.004 (from 0.076 to 0.072). Conversely, excluding ISVS6 resulted in a CFI increase of 0.015 (from 0.780 to 0.795) and an SRMR decrease of 0.020 (from 0.076 to 0.074). The exclusion of ISVS1 from the student dataset resulted in a 0.007 increase in CFI (from 0.834 to 0.841) and a 0.001 decrease in SRMR (from 0.062 to 0.061). Conversely, excluding ISVS6 led to CFI increasing by 0.012 points (from 0.834 to 0.846) and SRMR decreasing by 0.018 points (from 0.062 to 0.058). Other indices (i.e., CMIN/df and RMSEA) did not show improvement in both datasets. Similarly, excluding ISVS1 and ISVS6 in both datasets only introduced a minimal improvement in the fit indices.

For all of these trial results, the CMIN/df remained acceptable (between 3 and 5, excellent if <3), the CFI remained within the acceptable category (CFI not increased to >0.9), and the SRMR was already at an excellent level, as indicated by its initial value of <0.08. As such, the small improvement did not lead to better indices group classification compared to the initial model. Having an excellent SRMR (<0.08), acceptable CFI (>0.80), and acceptable CMIN/df (between 3—5) in both datasets already meets the COSMIN good model fit requirements. The slight improvement in the indices was deemed insignificant compared to the potential compromise of the instrument’s construct validity (i.e., if ISVS1, ISVS6, or both items were removed). Fig 2 shows changes in fit indices of the initial and final model for both datasets. Additional information regarding the CFA results is provided as a supplementary (S1 File).

As reflected in Fig 2 the model fit indices of the two cohorts improved significantly in the final model. Practitioner, *χ*^2^(187)=572.08, CMIN/df = 3.06, SRMR = 0.071, CFI = 0.834; and student, *χ*^2^(187)=723.43, CMIN/df = 3.87, SRMR = 0.059, CFI = 0.853. Both models met the COSMIN requirements for a good model fit. For a detailed comparison of the initial and final model fit indices, see also suplementary document for CFA results (S1 File). With a good model fit achieved, the MG-CFA for both datasets was confirmed to be appropriate for testing. Using the final model solutions, the standardised estimates, critical ratios and probability (*p*) for items in the two cohorts were calculated and presented in Table 3.

**Table 3:** Item estimates, critical ratios and probability (*p*).

Items	Practitioner				Student			
	Estimate^1^	S.E.^2^	C.R.^3^	*p*	Estimate^1^	S.E.^2^	C.R.^3^	*p*
ISVS1	0.53	0.13	6.24	<0.001	0.67	0.11	9.25	<0.001
ISVS2	0.58	0.13	7.00	<0.001	0.63	0.10	8.68	<0.001
ISVS3	0.62	0.11	7.33	<0.001	0.71	0.10	9.82	<0.001
ISVS4	0.80	0.13	9.44	<0.001	0.75	0.12	10.37	<0.001
ISVS5	0.76	0.13	8.90	<0.001	0.78	0.12	10.66	<0.001
ISVS6	0.72	0.17	8.56	<0.001	0.64	0.16	8.79	<0.001
ISVS7	0.72	0.15	8.58	<0.001	0.72	0.13	9.94	<0.001
ISVS8	0.76	0.14	8.97	<0.001	0.78	0.12	10.72	<0.001
ISVS9	0.68	0.12	7.90	<0.001	0.74	0.12	10.23	<0.001
ISVS10	0.78	0.14	9.33	<0.001	0.83	0.13	11.32	<0.001
ISVS11	0.64	0.12	7.37	<0.001	0.77	0.11	10.62	<0.001
ISVS12	0.81	0.12	9.59	<0.001	0.82	0.13	11.15	<0.001
ISVS13	0.70	0.10	8.03	<0.001	0.80	0.11	11.00	<0.001
ISVS14	0.62	0.13	7.16	<0.001	0.74	0.11	10.17	<0.001
ISVS15	0.82	0.13	9.61	<0.001	0.85	0.12	11.55	<0.001
ISVS16	0.67	0.10	7.68	<0.001	0.73	0.12	10.09	<0.001
ISVS17	0.71	0.13	8.38	<0.001	0.83	0.12	11.39	<0.001
ISVS18	0.80	0.14	9.41	<0.001	0.82	0.13	11.17	<0.001
ISVS19	0.73	0.11	8.50	<0.001	0.80	0.11	11.02	<0.001
ISVS20	0.76	0.13	8.77	<0.001	0.74	0.11	10.16	<0.001
ISVS21	0.75				0.69			

***Note***.

^1^Standardised estimates,

^2^Standard error,

^3^Critical ratio.

#### Measurement invariances

A 3-staged invariant measurement of configural, metric and scalar tests was carried out using the model presented in Fig 2B. Both datasets were analysed simultaneously. Thus, the reported fit indices refer to groups, not individual datasets. The configural invariance was achieved with the unconstrained model indicated SRMR = 0.059, RMSEA = 0.085, *χ*^2^(374)=1295.69, CMIN/df = 3.47, fulfilling the COSMIN criteria for a ‘good’ model fit [33]. The metric test indicated a good fit with SRMR = 0.061, RMSEA = 0.084, *χ*^2^(394)=1327.18, CMIN/df = 3.37, fulfilling the COSMIN criteria for a ‘good’ model fit [31]. Metric invariance was confirmed by the difference in CFI between configural and metrics models <0.01 (ΔCFI = 0.002). The scalar test indicated a good fit with SRMR = 0.061, RMSEA = 0.082, *χ*^2^(415)=1375.74, CMIN/df = 3.32, fulfilling the COSMIN criteria for a ‘good’ model fit [33]. Scalar invariance was confirmed by the difference in CFI between metrics and scalar models <0.01 (ΔCFI = 0.005). A comparison of fit indices for the three models is presented in Table 4.

**Table 4:** Full model comparison of the invariances.

Full Model Comparison	CMIN/df	CFI	ΔCFI	SRMR	RMSEA	Invariance
Configural Invariance	1295.69 (374) = 3.47	0.840	-	0.059	0.085	Yes
Metric Invariance (Measurement weights)	1327.18 (394) = 3.37	0.838	0.002	0.061	0.084	Yes
Scalar Invariance (Measurement intercepts)	1375.74 (415) = 3.32	0.833	0.005	0.061	0.082	Yes

***Note***. CMIN/df = Chi-square minimum discrepancy function, df = Degree of Freedom; CFI = comparative fit index, ΔCFI = differences in CFI, SRMR = standard root mean square of the residual, RMSEA = root mean square error of approximation.

#### Internal consistency reliability

The internal consistency reliabilities of the Australian ISVS-21 for practitioners and students indicated composite reliability of 0.96 and 0.96, respectively. The average variance extracted was within the expected range of greater than 0.5 (practitioner, AVE = 0.51; student, AVE = 0.57).

### Hypotheses testing

As neither dataset was normally distributed, non-parametric statistics of the Kruskal-Wallis H test were performed with the Mann-Whitney U posthoc test for comparisons to identify exact group differences. All responses (practitioners, *n*=134, student, *n*=207) were included for hypotheses testing.

**H1.A was accepted**. Practitioners’ age was a discriminant factor for interprofessional socialisation (*H*(4)=10.37, *p*=0.04). Practitioners aged 51–60 years were significantly different from those aged 21–30 years, 31–40 years, and 41–50 years.
**H2.A was accepted**. Practitioners’ length of service was a discriminant factor for interprofessional socialisation (*H*(6)=13.01, *p*=0.04). Practitioners with 1–2 years of work experience were significantly different from those with 11–15 years and 16–20 years; and those with 3–5 years of work experience were significantly different from those with 16–20 years, 21–30 years, and 30–40 years.
**H3.A was rejected**. Practitioners’ professional background was a discriminant factor for interprofessional socialisation for health practitioners (*H*(14)=28.20, *p*=0.01). Optometrists were significantly different from speech pathologists and social workers; psychologists were significantly different from podiatrists, social workers, speech pathologists, nurses, occupational therapists, pharmacists, nutritionists, medical practitioners, and physiotherapists; and dentists were significantly different from nurses and speech pathologists.
**H1.B was accepted**. Students’ age was a discriminant factor for interprofessional socialisation (*H*(2)=8.85, *p*=0.01). Students aged 35–40 were significantly different from those aged 18–24 and 25–34.
**H2.B was accepted**. Students’ length of study was a discriminant factor for interprofessional socialisation (*H*(3)=8.22, *p*=0.04). Students with 5–6 years of study were significantly different from those with 3–4 years and 7–8 years of study.
**H3.B was accepted**. Students’ discipline background was a discriminant factor for interprofessional socialisation (*H*(12)=11.98, *p*=0.45).

The post hoc tests with Mann-Whitney U identified significant differences related to exact age groups for each hypothesis (please refer to supplementary S2 Table). Five of the six (83.3%) assumptions proposed were accepted; therefore, COSMIN’s requirements for hypotheses testing were met [33].

## Discussion

This study aimed to assess the psychometric properties of the Australian ISVS. A series of psychometric evaluations were carried out according to COSMIN guidelines, which included content validity testing, internal structure testing (structural validity, internal consistency reliability, and measurement invariance), and hypotheses testing. In particular, measurement invariance was assessed to ascertain instrument invariance across tested groups and suitability for use by health practitioners and students in Australia.

### The Australian ISVS-21 psychometric properties

The Australian ISVS was confirmed to be unidimensional, suggesting that all 21 items that comprise the instrument measure a similar construct of interprofessional socialisation as proposed by the original instrument [9]. Several studies in various countries have also confirmed the unidimensional structure of ISVS-21 [15, 19, 36].

Based on content validity analysis for COSMIN requirements on *relevance*, *comprehensibility* and *comprehensiveness*, no changes were made, and all 21 items were used as presented in the original version [9]. However, it should be noted that four items were closely examined in the pilot study in terms of how easily they could be understood (comprehensibility): ISVS2 (better appreciation for using a common language), ISVS4 (able to share and exchange ideas), ISVS5 (enhanced perception of engaging in interprofessional practice), and ISVS15 (comfortable clarifying misconceptions). Although participants suggested alternative wording for these items, the research panel ultimately retained the original language as the original items aligned better with the intended constructs. ISVS2 and ISVS5 were also recommended to be rewritten in other validation studies [8, 19], whereas ISVS15 was removed from the other two studies to improve the instrument’s properties [8, 14].

In addition, three items received the least endorsement in terms of *relevance* from both cohorts in the pilot study: ISVS1 (aware of preconceived ideas), ISVS6 (comfortable being the leader), and ISVS7 (comfortable in speaking out). The ISVS1 and ISVS6 are of concern because subsequent confirmatory factor analysis also confirmed ISVS1 (aware of preconceived ideas) as the item with the lowest estimate in the practitioner cohort and ISVS6 (comfortable being the leader) as the second lowest in the student cohort. This finding is consistent with results demonstrated during the development of ISVS-21 [9], whereby ISVS6 (comfortable being the leader) and ISVS1 (aware of preconceived ideas) were the two items with the lowest means. Similar findings related to difficulties with ISVS1 (aware of preconceived ideas) were corroborated by other ISVS-21 studies using student participants in Germany [15], Spain [19], Australia [27], and Indonesia [36]; and ISVS6 (comfortable being the leader) was dropped from the instrument in the study in Australia [27]. A low loading estimate on an item indicates a weak contribution of the item to the overall construct [58]. Collectively, these results reinforce the weaknesses of the two items for inclusion in the measure.

The internal consistency reliability scores of the Australian ISVS-21 are relatively high, with Cronbach’s *α* scores and composite reliability exceeding 0.95 for both datasets. This suggests that there may be item redundancy. The interitem correlation scores in both datasets also exceed 0.5, indicating further redundancy. These reliability scores demonstrate how well the items are related to each other and their suitability for measuring a single construct. While high reliability is essential, excessively high scores may compromise the instrument’s ability to measure diverse constructs accurately, thus impacting its overall validity [33, 63].

Cronbach’s *α* scores of well above 0.90 have been reported in several ISVS-21 studies [15, 19, 30] and some with very high scores of >0.95 [22, 26], including the original ISVS-21 with Cronbach’s *α*=0.988 [9]. Item reduction is recommended for a scale or subscale with very high Cronbach’s *α* scores [33]. While our psychometric evaluation of the Australian ISVS-21 indicates that attention needs to be paid to their relevance for inclusion in the instrument to ISVS1 (aware of preconceived ideas) and ISVS6 (comfortable being the leader), relying on Cronbach’s *α* or composite reliability, and interitem correlation scores alone as a basis for item deletion is not recommended at this stage. Instead, all 21 items were seen to have relevance, were well-understood, and were comprehensive for measuring interprofessional socialisation in Australia. As good psychometric properties of the instrument can still be maintained by retaining these items, retaining ISVS1 (aware of preconceived ideas) and ISVS6 (comfortable being the leader) is highly recommended to allow measuring themes that are challenging, yet highly relevant, to interprofessional education and collaborative practice.

The three-stage invariance tests of configural, metric and scalar performed on the Australian ISVS confirmed the proposed model is invariant across groups tested and can be used to measure interprofessional socialisation in Australian health practitioners and students. By achieving configuration, metric, and scalar invariants, practitioners and students are in agreement with the Australian ISVS factorial structure regarding its unidimensionality and inclusion of all 21 items [65, 66]. The practitioners and students in the study shared the same understanding of the constructs that underlie the Australian ISVS, and the mean scores of the two cohorts are expected to be comparable when assessed using the suggested factorial solutions [65, 66].

To date, the only studies we found to have evaluated the equivalence of the ISVS-21 for use in practitioners and students was the original Canadian version [9]. The study used Item Response Theory modelling and reported an agreement score between the practitioner and student datasets (r = 0.9986, 95% CI 0.9983–0.9988), indicating invariance of the two tested cohorts. However, because this original study used a different invariant measurement method to the ones performed in this study, it should be interpreted with caution.

### ‘Preconceived ideas’ and ‘leadership’ in interprofessional socialisation

Participant disagreement over including two items, ISVS1 (aware of preconceived ideas) and ISVS6 (comfortable being the leader), reflects a challenge to the concepts implied by the items, which are *preconceived ideas* and *leadership* in interprofessional socialisation. Both practitioners and students involved in this research have experience working in interprofessional teams. This experience developed their perceptions, attitudes, and beliefs about interprofessional teamwork and shaped their preconceived ideas. Stereotypes about one’s role, the roles of other team members, and the dominance of certain professions are among the frequently expressed prejudices [4, 67, 68], which were evident in these studies.

Leadership is an important element of effective interprofessional teamwork [1, 10]. Leadership is regarded as an antecedent factor that can directly or indirectly affect consequence variables [10], such as conflict resolution [69, 70], team effectiveness [23, 24], the team’s drive for patient involvement [25, 70], and patient satisfaction [68, 71]. The fact that ISVS6 (comfortable being the leader) is not fully supported in many ISVS studies suggests challenges for leadership skills in interprofessional teams, which align with previous research on the complexity of interprofessional practice [67].

It is worth noting that women were the dominant participants in both the pilot and validation studies. According to the World Health Organization [72], women comprise almost 70% of global health workers, with 89% being nurses. This trend is also reflected in Australia, where women comprise 74.2% of the healthcare workforce [73]. Nurses and midwives constitute Australia’s largest group of registered health professionals, representing about 54% of the total health professionals. Through a purposive sampling method, we distributed the instrument online to health professional bodies and other official platforms freely accessible to healthcare practitioners and students in Australia. As a result, the overrepresentation of women in the Australian healthcare workforce naturally led to recruiting more female than male participants.

The significant number of women in this study could potentially result in implicit bias. Participants’ experiences may have influenced their preconceived ideas about prevalent issues impacting women as healthcare professionals, ranging from a simple stereotypical based on gender characteristics, as to how male and female will act in the group, to more complex issues, including gender disparities, work-life balance and family responsibilities, unfavourable work environments, and discrimination (e.g., limited job recruitment opportunities and career advancement for women) [74, 75]. These experiences may have shaped the participants’ views on interprofessional socialisation and values.

Despite the high percentage of women in clinical and frontline healthcare roles in Australia, their presence in leadership positions does not reflect this. Only 12.5% of hospital chief executive officers, 22% of Australian Medical Association presidents, 28% of medical school deans, and 33% of chief medical officers at state/territory and federal levels are women [76]. Barriers such as self-doubt, lack of self-confidence, underestimating personal capabilities, parenthood or family responsibilities, and the perception that being too feminine means being an incompetent leader prevent women from taking up leadership roles [75–79]. More female representation in leadership roles is urgently needed to ensure inclusive decision-making for women in Australia’s health sector.

### Determining factors for interprofessional socialisation

In relation to construct validity, this study confirms that age and length of service or length of study were determining factors for interprofessional socialisation for both health practitioners and students. Age and years of service have been acknowledged as influential factors for interprofessional practice [37, 38]. The two are related because older practitioners with more extensive work experience will likely understand their scope of practice better [41, 43, 45]. However, previous research found that more experienced practitioners are less likely to collaborate and share information [37, 45]. Furthermore, given that professional role identity occurs at least six months after the start of clinical exposure [44], it was not surprising that students’ discipline-specific skills begin to shape during their clinical placement time and, subsequently, their professional identity in general. The students who participated in this study had a minimum of one year of clinical experience.

Students’ course of study was not a discriminant factor for interprofessional socialisation, which is consistent with other previous ISVS studies identifying no significant differences between students regarding study courses [14, 16–18, 20, 22]. Studies using the ISVS with practitioners are limited. However, many studies have reported that professional background is a differentiating factor, and professional hierarchies are evident in healthcare [45, 47, 48].

Interestingly, professional backgrounds were evident for practitioners but not for students. One possible explanation is that the Australian students involved have been trained in IPE during their training and thus were more accepting of interprofessional socialisation [4]. In contrast, the practitioners in this study, 33% of whom have been in service for over 15 years, are likely to have been trained in uniprofessional culture and practice [51, 52] as IPE is a relatively new addition to health professional education. As such, they may be less inclined to collaborate with other practitioners in patient care [4, 43].

This study has limitations. The samples were dominated by certain professions or courses, which may have influenced participants’ overall responses. For future action, research utilising item response theory (Rasch analysis) is needed to determine if the problematic items (i.e., ISVS1 and ISVS6) need to be removed.

## Conclusion

The Australian ISVS has good psychometric properties based on evaluating the content validity, internal structure, and hypotheses testing for construct validity. In addition, Australian ISVS is an invariant measure for use by health practitioners and students and, therefore, is confirmed as a quality measure to assess interprofessional socialisation for both cohorts in Australia.

## Supporting information

S1 Table Pilot participants characteristics. (DOCX)

S2 Table Mann-Whitney U post-hoc analysis of significant hypotheses testing. (DOCX)

S1 File Detailed CFA results. (DOCX)
