# Supplementary material for: Psychometric evaluation of the Australian interprofessional socialisation and valuing scale: An invariant measure for health practitioners and students
Source: PLoS One. 2024 Sep 6;19(9):e0309697. doi: 10.1371/journal.pone.0309697 (PMC11379266; doi:10.1371/journal.pone.0309697)
Supplement: S1 Table — (DOCX) [file pone.0309697.s001.docx]

**S1 Table.** Pilot participants characteristics

|  |  |  |  |  |  |  |  |
| --- | --- | --- | --- | --- | --- | --- | --- |
| **Practitioners (n = 23)** | | | | **Students (n = 9)** | | | |
| **Demographics** | **Frequency (%)** | **Mean** | **SD** | **Demographics** | **Frequency (%)** | **Mean** | **SD** |
| **Gender** | | | | | | | |
| Male | 5 (21.7%) | - | - | Male | 0 (0%) | - | - |
| Female | 18 (78.3%) |  |  | Female | 9 (100%) |  |  |
| Total | 23 (100%) |  |  | Total | 9 (100%) |  |  |
| **Age** | | | | | | | |
| 21-25 years | 1 (4.3%) | 39.3 | 8.7 | 18-24 years | 5 (55.6%) | 27 | 7.3 |
| 26-30 years | 4 (17.4%) |  |  | 25-29 years | 1 (11.1%) |  |  |
| 31-35 years | 2 (8.7%) |  |  | 30-34 years | 0 (0%) |  |  |
| 36-40 years | 5 (21.7%) |  |  | 35-40 years | 3 (33.3%) |  |  |
| 41-45 years | 7 (30.4%) |  |  | Total | 9 (100%) |  |  |
| 46-50 years | 1 (4.3%) |  |  |  |  |  |  |
| 51-55 years | 2 (8.7%) |  |  |  |  |  |  |
| 56-60 years | 1 (4.3%) |  |  |  |  |  |  |
| Total | 23 (100%) |  |  |  |  |  |  |
| **Length of Work/Length of Study** | | | | | | | |
| 1-2 years | 7 (30.4%) | 9.2 | 8.5 | 1-2 years | 2 (22.2%) | 3.0 | 0.9 |
| 3-5 years | 4 (17.4%) |  |  | 3-4 years | 7 (77.8%) |  |  |
| 6-10 years | 4 (17.4%) |  |  | Total | 9 (100%) |  |  |
| 11-15 years | 4 (17.4%) |  |  |  | | | |
| 21-30 years | 4 (17.4%) |  |  |  |  |  |  |
| Total | 23 (100%) |  |  |  |  |  |  |
| **Professional/Educational Backgrounds** | | | | | | | |
| Medical practitioner | 1 (4.3%) | - | - | Medicine | 2 (22.2%) | - | - |
| Midwife | 1 (4.3%) |  |  | Nursing | 1 (11.1%) |  |  |
| Nurse | 11 (47.8) |  |  | Occupational therapy | 5 (55.6%) |  |  |
| Pharmacist | 1 (4.3%) |  |  | Dentistry | 1 (11.1%) |  |  |
| Physiotherapist | 1 (4.3%) |  |  | Total | 9 (100%) |  |  |
| Public health expert | 1 (4.3%) |  |  |  | | | |
| Speech pathologist | 5 (21.7%) |  |  |  |  |  |  |
| Social Workers | 2 (8.7%) |  |  |  |  |  |  |
| Total | 23 (100%) |  |  |  |  |  |  |
