# Supplementary material for: Psychometric evaluation of the Australian interprofessional socialisation and valuing scale: An invariant measure for health practitioners and students
Source: PLoS One. 2024 Sep 6;19(9):e0309697. doi: 10.1371/journal.pone.0309697 (PMC11379266; doi:10.1371/journal.pone.0309697)
Supplement: S2 Table — (DOCX) [file pone.0309697.s002.docx]

**S3 Table. Mann-Whitney U post-hoc analysis of significant hypotheses testing**

| **Practitioners** | | | | | | **Students** | | | | | |
| --- | --- | --- | --- | --- | --- | --- | --- | --- | --- | --- | --- |
| **Demographics** | | **Mann-Whitney U** | **Z** | ***p*** | ***r*** | **Demographics** | | **Mann-Whitney U** | **Z** | ***p*** | ***r*** |
| **Age** | | | | | | **Age** | | | | | |
| 51-60 years (*Md*=112, *n*=15) | 21-30 years (*Md*=93.0, *n*=31) | 103.00 | -3.04 | 0.002 | 0.45 | 35-40 years (*Md*=83, *n*=11) | 18-24 years (*Md*=100.5, *n*=150) | 481.00 | -2.31 | 0.021 | 0.18 |
|  | 31-40 years (*Md*=114.5, *n*=56) | 271.00 | -2.10 | 0.036 | 0.25 |  | 25-34 years (*Md*=104.0, *n*=46) | 112.00 | -2.85 | 0.004 | 0.38 |
|  | 41-50 years (*Md*=94.0, *n*=26) | 100.50 | -2.56 | 0.010 | 0.40 |  | | | | | |
| **Length of service** | | | | | | **Length of Study** | | | | | |
| 1-2 years (*Md*=93, *n*=22) | 16-20 years (*Md*=113.5, *n*=12) | 73.00 | -2.13 | 0.033 | 0.37 | 5-6 years (*Md*=91.50, *n*=10) | 3-4 years *(Md*=102.0, *n*=73) | 190.50 | -2.00 | 0.046 | 0.23 |
|  | 11-15 years (*Md*=94.0, *n*=19) | 98.00 | -2.22 | 0.026 | 0.37 |  | 7-8 years (*Md*=117.50, *n*=12) | 0.00 | -2.15 | 0.032 | 0.62 |
| 3-5 years (*Md*=94, *n*=18) | 16-20 years (*Md*=113.5, *n*=21) | 41.00 | -2.84 | 0.005 | 0.52 |  | | | | | |
|  | 21-30 years (*Md*=94.0, *n*=18) | 106.50 | -2.33 | 0.020 | 0.37 |  |  |  |  |  |  |
|  | 30-40 years (*Md*=109.0, *n*=11) | 38.50 | -2.72 | 0.007 | 0.51 |  |  |  |  |  |  |
| **Professional Backgrounds** | | | | | |  |  |  |  |  |  |
| Optometrist (*Md*= 86.0, *n*=6) | Speech pathologists (*Md*= 105.0, *n*=23) | 26.00 | -2.32 | 0.020 | 0.43 |  |  |  |  |  |  |
|  | Social Workers (*Md*= 117.5, *n*=4) | 2.00 | -2.15 | 0.032 | 0.68 |  |  |  |  |  |  |
| Psychologists (*Md*=77.00, *n*=4) | Podiatrists (*Md*= 123.5, *n*=2) | 0.00 | -2.00 | 0.046 | 0.71 |  |  |  |  |  |  |
|  | Social Workers (*Md*= 117.5, *n*=4) | 0.00 | -2.31 | 0.021 | 0.82 |  |  |  |  |  |  |
|  | Speech pathologists (*Md*= 105.0, *n*=23) | 3.00 | -2.94 | 0.003 | 0.57 |  |  |  |  |  |  |
|  | Nurses (*Md*= 106.0, *n*=18) | 7.00 | -2.47 | 0.014 | 0.53 |  |  |  |  |  |  |
|  | Occupational therapists (*Md*= 102.5, *n*=30) | 15.00 | -2.41 | 0.016 | 0.41 |  |  |  |  |  |  |
|  | Pharmacists (*Md*= 90.0, *n*=15) | 10.00 | -2.01 | 0.044 | 0.46 |  |  |  |  |  |  |
|  | Nutritionists (*Md*= 104.0, *n*=3) | 0.00 | -2.12 | 0.034 | 0.80 |  |  |  |  |  |  |
|  | Medical practitioners (*Md*= 104.0, *n*=10) | 2.50 | -2.48 | 0.013 | 0.66 |  |  |  |  |  |  |
|  | Physiotherapists (*Md*= 98.0, *n*=7) | 0.00 | -2.06 | 0.040 | 0.69 |  |  |  |  |  |  |
| Dentists (*Md*=72.50, *n*=2) | Nurses (*Md*= 106.0, *n*= 18) | 2.00 | -2.02 | 0.044 | 0.45 |  |  |  |  |  |  |
|  | Speech pathologists (*Md*= 105.0, *n*= 23) | 1.00 | -2.21 | 0.027 | 0.44 |  |  |  |  |  |  |
