# Supplementary material for: Psychometric evaluation of the Australian interprofessional socialisation and valuing scale: An invariant measure for health practitioners and students
Source: PLoS One. 2024 Sep 6;19(9):e0309697. doi: 10.1371/journal.pone.0309697 (PMC11379266; doi:10.1371/journal.pone.0309697)
Supplement: S1 File — (DOCX) [file pone.0309697.s003.docx]

**S2 File.** Detailed CFA results

| **Practitioner** | **Student** |
| --- | --- |
| **Initial CFA model with fit indices**  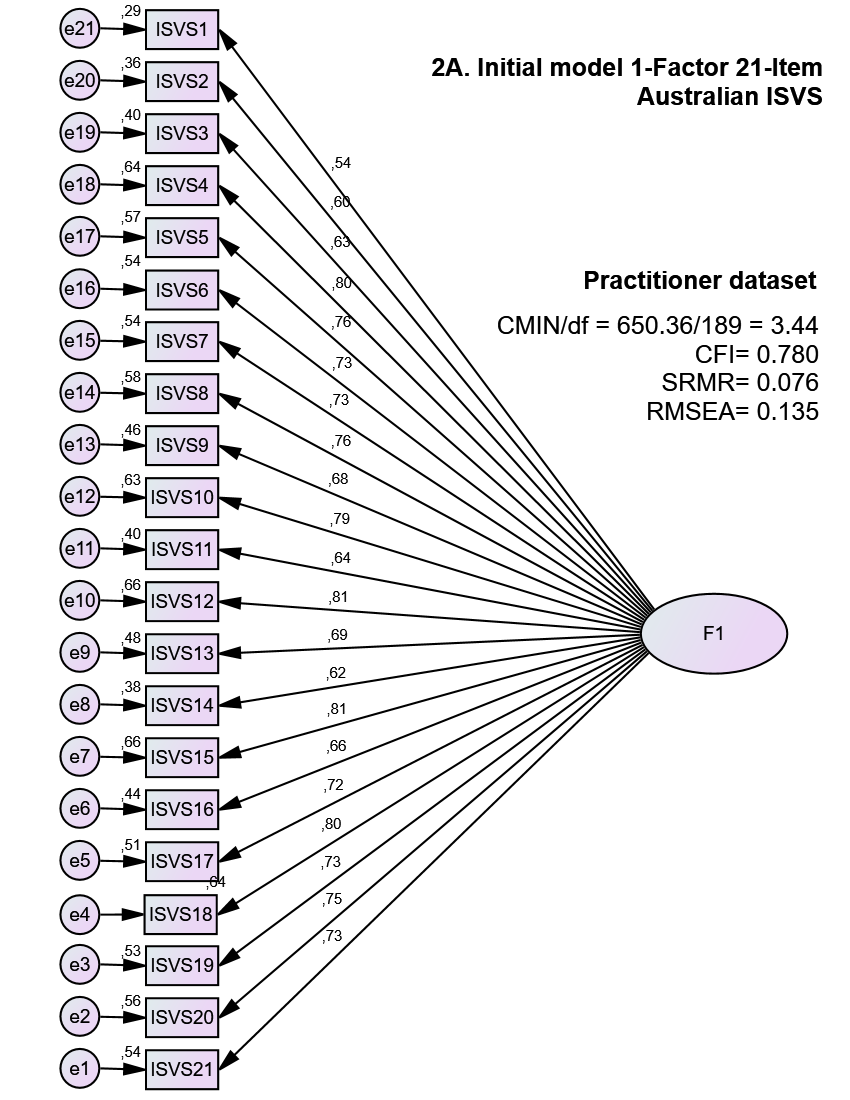 | **Initial CFA dataset with fit indices**  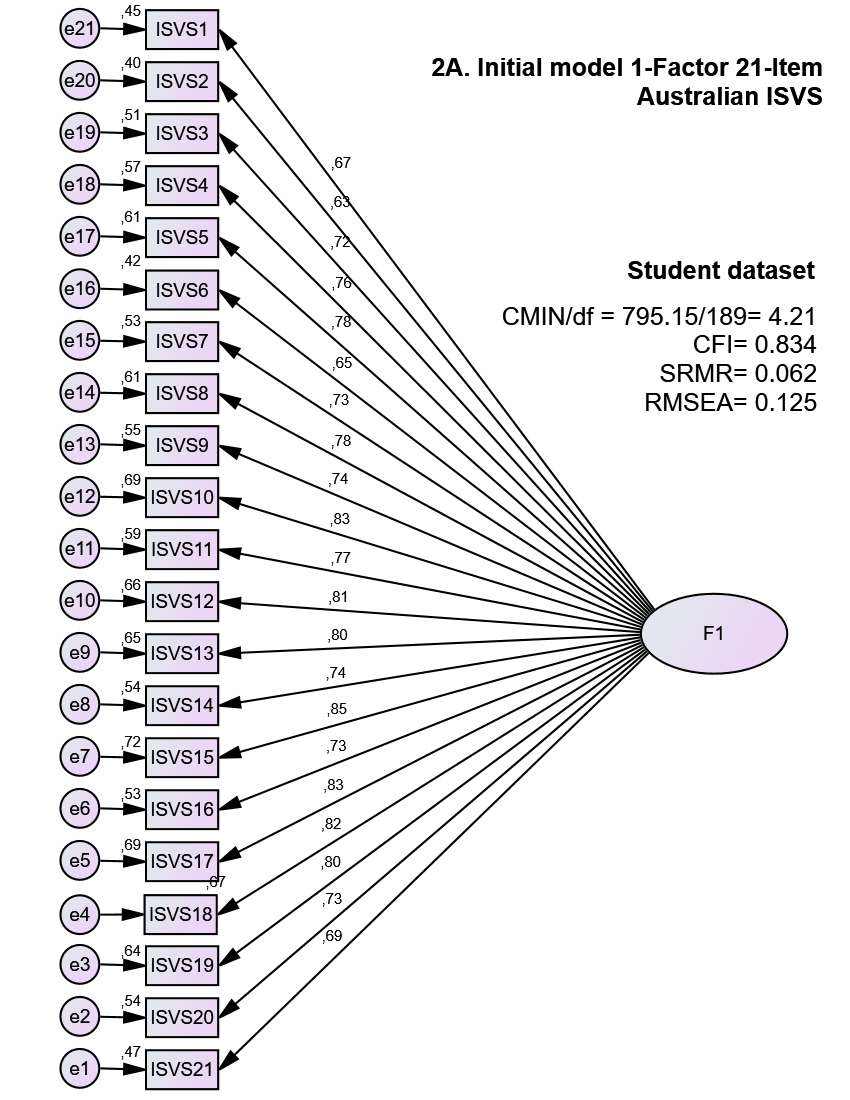 |
| **Initial Standardised Regression Weights**   \|  \|  \|  \| **Estimate^1^** \| \| **S.E.^2^** \| \| **C.R.^3^** \| \| ***p^4^*** \|  \| \| \| \| --- \| --- \| --- \| --- \| --- \| --- \| --- \| --- \| --- \| --- \| --- \| --- \| --- \| \| ISVS21 \| <-- \| F1 \| 0.731 \|  \| \|  \| \|  \| \| \|  \| \| ISVS20 \| <-- \| F1 \| 0.748 \| .131 \| \| 8.769 \| \| < 0.001 \| \| \|  \| \| \| \| ISVS19 \| <-- \| F1 \| 0.727 \| .109 \| \| 8.497 \| \| < 0.001 \| \| \|  \| \| \| \| ISVS17 \| <-- \| F1 \| 0.717 \| .128 \| \| 8.375 \| \| < 0.001 \| \| \|  \| \| \| \| ISVS16 \| <-- \| F1 \| 0.661 \| .103 \| \| 7.684 \| \| < 0.001 \| \| \|  \| \| \| \| ISVS15 \| <-- \| F1 \| 0.815 \| .133 \| \| 9.608 \| \| < 0.001 \| \| \|  \| \| \| \| ISVS14 \| <-- \| F1 \| 0.618 \| .130 \| \| 7.157 \| \| < 0.001 \| \| \|  \| \| \| \| ISVS13 \| <-- \| F1 \| 0.690 \| .096 \| \| 8.034 \| \| < 0.001 \| \| \|  \| \| \| \| ISVS12 \| <-- \| F1 \| 0.813 \| .121 \| \| 9.587 \| \| < 0.001 \| \| \|  \| \| \| \| ISVS11 \| <-- \| F1 \| 0.636 \| .119 \| \| 7.371 \| \| < 0.001 \| \| \|  \| \| \| \| ISVS10 \| <-- \| F1 \| 0.793 \| .140 \| \| 9.328 \| \| < 0.001 \| \| \|  \| \| \| \| ISVS9 \| <-- \| F1 \| 0.678 \| .124 \| \| 7.895 \| \| < 0.001 \| \| \|  \| \| \| \| ISVS8 \| <-- \| F1 \| 0.765 \| .138 \| \| 8.973 \| \| < 0.001 \| \| \|  \| \| \| \| ISVS7 \| <-- \| F1 \| 0.734 \| .145 \| \| 8.582 \| \| < 0.001 \| \| \|  \| \| \| \| ISVS5 \| <-- \| F1 \| 0.758 \| .128 \| \| 8.892 \| \| < 0.001 \| \| \|  \| \| \| \| ISVS4 \| <-- \| F1 \| 0.802 \| .134 \| \| 9.442 \| \| < 0.001 \| \| \|  \| \| \| \| ISVS3 \| <-- \| F1 \| 0.632 \| .113 \| \| 7.328 \| \| < 0.001 \| \| \|  \| \| \| \| ISVS2 \| <-- \| F1 \| 0.603 \| .125 \| \| 6.980 \| \| < 0.001 \| \| \|  \| \| \| \| ISVS1 \| <-- \| F1 \| 0.542 \| .130 \| \| 6.236 \| \| < 0.001 \| \| \|  \| \| \| \| ISVS6 \| <-- \| F1 \| 0.732 \| .168 \| \| 8.564 \| \| < 0.001 \| \| \|  \| \| \| \| ISVS18 \| <-- \| F1 \| 0.799 \| .137 \| \| 9.407 \| \| < 0.001 \| \| \|  \| \| \|   **Notes.** ^1^Standardised estimates; ^2^Standar Error; ^3^Critical ratio; ^4^Significant at 95% CI. | **Initial Standardised Regression Weights**   \|  \|  \|  \| **Estimate^1^** \| **S.E.^2^** \| **C.R.^3^** \| ***p*** \| \| --- \| --- \| --- \| --- \| --- \| --- \| --- \| \| ISVS21 \| <-- \| F1 \| 0.686 \|  \|  \|  \| \| ISVS20 \| <-- \| F1 \| 0.734 \| .109 \| 10.024 \| < 0.001 \| \| ISVS19 \| <-- \| F1 \| 0.801 \| .112 \| 10.870 \| < 0.001 \| \| ISVS17 \| <-- \| F1 \| 0.831 \| .125 \| 11.250 \| < 0.001 \| \| ISVS16 \| <-- \| F1 \| 0.728 \| .118 \| 9.942 \| < 0.001 \| \| ISVS15 \| <-- \| F1 \| 0.846 \| .125 \| 11.429 \| < 0.001 \| \| ISVS14 \| <-- \| F1 \| 0.736 \| .110 \| 10.054 \| < 0.001 \| \| ISVS13 \| <-- \| F1 \| 0.803 \| .110 \| 10.903 \| < 0.001 \| \| ISVS12 \| <-- \| F1 \| 0.813 \| .129 \| 11.021 \| < 0.001 \| \| ISVS11 \| <-- \| F1 \| 0.771 \| .111 \| 10.498 \| < 0.001 \| \| ISVS10 \| <-- \| F1 \| 0.831 \| .135 \| 11.241 \| < 0.001 \| \| ISVS9 \| <-- \| F1 \| 0.745 \| .118 \| 10.163 \| < 0.001 \| \| ISVS8 \| <-- \| F1 \| 0.783 \| .118 \| 10.641 \| < 0.001 \| \| ISVS7 \| <-- \| F1 \| 0.730 \| .134 \| 9.967 \| < 0.001 \| \| ISVS5 \| <-- \| F1 \| 0.779 \| .118 \| 10.601 \| < 0.001 \| \| ISVS4 \| <-- \| F1 \| 0.758 \| .121 \| 10.329 \| < 0.001 \| \| ISVS3 \| <-- \| F1 \| 0.717 \| .099 \| 9.805 \| < 0.001 \| \| ISVS2 \| <-- \| F1 \| 0.634 \| .104 \| 8.730 \| < 0.001 \| \| ISVS1 \| <-- \| F1 \| 0.674 \| .113 \| 9.249 \| < 0.001 \| \| ISVS6 \| <-- \| F1 \| 0.647 \| .160 \| 8.903 \| < 0.001 \| \| ISVS18 \| <-- \| F1 \| 0.816 \| .134 \| 11.056 \| < 0.001 \|   **Notes.** ^1^Standardised estimates; ^2^Standar Error; ^3^Critical ratio; ^4^Significant at 95% CI |
| **Final CFA model with fit indices**  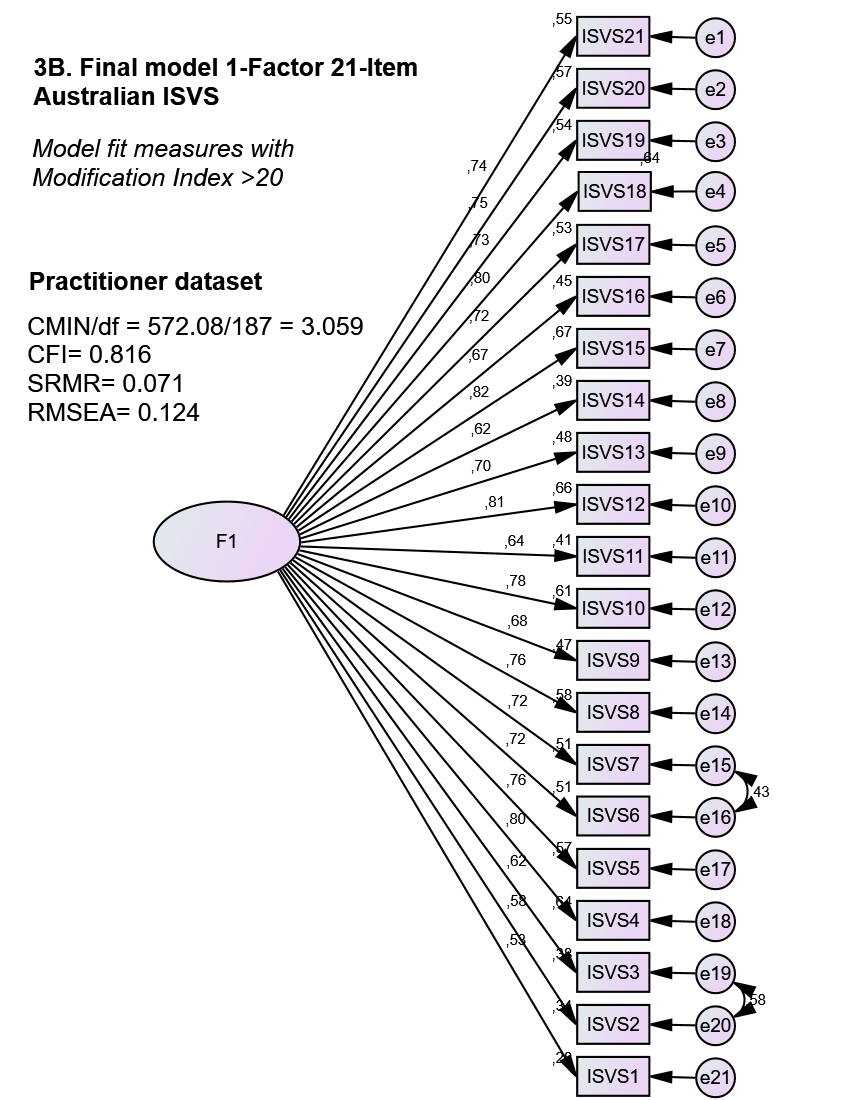 | **Final CFA model with fit indices**  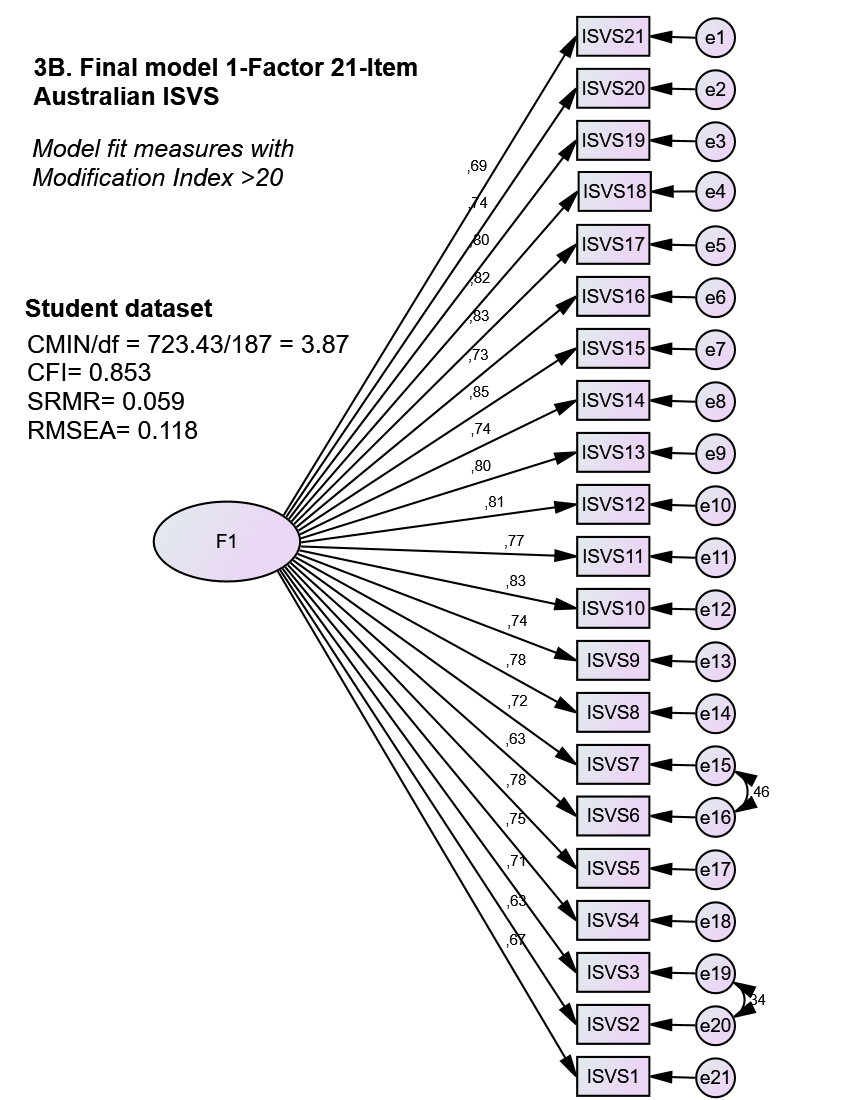 |
| **Final Standardised Regression Weights**   \|  \|  \|  \| **Estimate^1^** \| \| **S.E.^2^** \| \| **C.R.^3^** \| \| ***p^4^*** \|  \| \| \| \| --- \| --- \| --- \| --- \| --- \| --- \| --- \| --- \| --- \| --- \| --- \| --- \| --- \| \| ISVS21 \| <-- \| F1 \| 0.744 \|  \| \|  \| \|  \| \| \|  \| \| ISVS20 \| <-- \| F1 \| 0.755 \| 0.127 \| \| 9.014 \| \| < 0.001 \| \| \|  \| \| \| \| ISVS19 \| <-- \| F1 \| 0.732 \| 0.105 \| \| 8.713 \| \| < 0.001 \| \| \|  \| \| \| \| ISVS17 \| <-- \| F1 \| 0.725 \| 0.124 \| \| 8.617 \| \| < 0.001 \| \| \|  \| \| \| \| ISVS16 \| <-- \| F1 \| 0.668 \| 0.100 \| \| 7.876 \| \| < 0.001 \| \| \|  \| \| \| \| ISVS15 \| <-- \| F1 \| 0.817 \| 0.127 \| \| 9.855 \| \| < 0.001 \| \| \|  \| \| \| \| ISVS14 \| <-- \| F1 \| 0.621 \| 0.127 \| \| 7.276 \| \| < 0.001 \| \| \|  \| \| \| \| ISVS13 \| <-- \| F1 \| 0.696 \| 0.093 \| \| 8.236 \| \| < 0.001 \| \| \|  \| \| \| \| ISVS12 \| <-- \| F1 \| 0.811 \| 0.116 \| \| 9.769 \| \| < 0.001 \| \| \|  \| \| \| \| ISVS11 \| <-- \| F1 \| 0.639 \| 0.116 \| \| 7.503 \| \| < 0.001 \| \| \|  \| \| \| \| ISVS10 \| <-- \| F1 \| 0.784 \| 0.135 \| \| 9.400 \| \| < 0.001 \| \| \|  \| \| \| \| ISVS9 \| <-- \| F1 \| 0.683 \| 0.120 \| \| 8.077 \| \| < 0.001 \| \| \|  \| \| \| \| ISVS8 \| <-- \| F1 \| 0.762 \| 0.133 \| \| 9.107 \| \| < 0.001 \| \| \|  \| \| \| \| ISVS7 \| <-- \| F1 \| 0.717 \| 0.140 \| \| 8.507 \| \| < 0.001 \| \| \|  \| \| \| \| ISVS5 \| <-- \| F1 \| 0.756 \| 0.123 \| \| 8.892 \| \| < 0.001 \| \| \|  \| \| \| \| ISVS4 \| <-- \| F1 \| 0.801 \| 0.129 \| \| 9.442 \| \| < 0.001 \| \| \|  \| \| \| \| ISVS3 \| <-- \| F1 \| 0.617 \| 0.110 \| \| 7.328 \| \| < 0.001 \| \| \|  \| \| \| \| ISVS2 \| <-- \| F1 \| 0.584 \| 0.122 \| \| 6.980 \| \| < 0.001 \| \| \|  \| \| \| \| ISVS1 \| <-- \| F1 \| 0.530 \| 0.127 \| \| 6.236 \| \| < 0.001 \| \| \|  \| \| \| \| ISVS6 \| <-- \| F1 \| 0.716 \| 0.163 \| \| 8.564 \| \| < 0.001 \| \| \|  \| \| \| \| ISVS18 \| <-- \| F1 \| 0.800 \| 0.132 \| \| 9.407 \| \| < 0.001 \| \| \|  \| \| \|   **Notes.** ^1^Standardised estimates; ^2^Standar Error; ^3^Critical ratio; ^4^Significant at 95% CI. | **Final Standardised Regression Weights**   \|  \|  \|  \| **Estimate^1^** \| \| **S.E.^2^** \| \| **C.R.^3^** \| \| ***p^4^*** \|  \| \| \| \| --- \| --- \| --- \| --- \| --- \| --- \| --- \| --- \| --- \| --- \| --- \| --- \| --- \| \| ISVS21 \| <-- \| F1 \| 0.692 \|  \| \|  \| \|  \| \| \|  \| \| ISVS20 \| <-- \| F1 \| 0.738 \| 0.107 \| \| 10.156 \| \| < 0.001 \| \| \|  \| \| \| \| ISVS19 \| <-- \| F1 \| 0.804 \| 0.110 \| \| 11.018 \| \| < 0.001 \| \| \|  \| \| \| \| ISVS17 \| <-- \| F1 \| 0.833 \| 0.123 \| \| 11.388 \| \| < 0.001 \| \| \|  \| \| \| \| ISVS16 \| <-- \| F1 \| 0.733 \| 0.116 \| \| 10.087 \| \| < 0.001 \| \| \|  \| \| \| \| ISVS15 \| <-- \| F1 \| 0.846 \| 0.123 \| \| 11.553 \| \| < 0.001 \| \| \|  \| \| \| \| ISVS14 \| <-- \| F1 \| 0.739 \| 0.109 \| \| 10.168 \| \| < 0.001 \| \| \|  \| \| \| \| ISVS13 \| <-- \| F1 \| 0.803 \| 0.108 \| \| 10.997 \| \| < 0.001 \| \| \|  \| \| \| \| ISVS12 \| <-- \| F1 \| 0.815 \| 0.127 \| \| 11.154 \| \| < 0.001 \| \| \|  \| \| \| \| ISVS11 \| <-- \| F1 \| 0.774 \| 0.109 \| \| 10.624 \| \| < 0.001 \| \| \|  \| \| \| \| ISVS10 \| <-- \| F1 \| 0.828 \| 0.132 \| \| 11.323 \| \| < 0.001 \| \| \|  \| \| \| \| ISVS9 \| <-- \| F1 \| 0.743 \| 0.116 \| \| 10.226 \| \| < 0.001 \| \| \|  \| \| \| \| ISVS8 \| <-- \| F1 \| 0.781 \| 0.116 \| \| 10.718 \| \| < 0.001 \| \| \|  \| \| \| \| ISVS7 \| <-- \| F1 \| 0.721 \| 0.132 \| \| 9.9380 \| \| < 0.001 \| \| \|  \| \| \| \| ISVS5 \| <-- \| F1 \| 0.777 \| 0.116 \| \| 10.660 \| \| < 0.001 \| \| \|  \| \| \| \| ISVS4 \| <-- \| F1 \| 0.754 \| 0.119 \| \| 10.368 \| \| < 0.001 \| \| \|  \| \| \| \| ISVS3 \| <-- \| F1 \| 0.712 \| 0.098 \| \| 9.8170 \| \| < 0.001 \| \| \|  \| \| \| \| ISVS2 \| <-- \| F1 \| 0.627 \| 0.102 \| \| 8.6840 \| \| < 0.001 \| \| \|  \| \| \| \| ISVS1 \| <-- \| F1 \| 0.669 \| 0.111 \| \| 9.2470 \| \| < 0.001 \| \| \|  \| \| \| \| ISVS6 \| <-- \| F1 \| 0.635 \| 0.157 \| \| 8.7940 \| \| < 0.001 \| \| \|  \| \| \| \| ISVS18 \| <-- \| F1 \| 0.816 \| 0.132 \| \| 11.169 \| \| < 0.001 \| \| \|  \| \| \|   **Notes.** ^1^Standardised estimates; ^2^Standar Error; ^3^Critical ratio; ^4^Significant at 95% CI. |
